# Parental knowledge, attitudes and practices regarding antibiotic use for acute upper respiratory tract infections in children: a cross-sectional study in Palestine

**DOI:** 10.1186/s12887-015-0494-5

**Published:** 2015-11-11

**Authors:** Sa’ed H. Zyoud, Adham Abu Taha, Khulood F. Araj, Islam A. Abahri, Ansam F. Sawalha, Waleed M. Sweileh, Rahmat Awang, Samah W. Al-Jabi

**Affiliations:** Poison Control and Drug Information Center (PCDIC), College of Medicine and Health Sciences, An-Najah National University, Nablus, 44839 Palestine; Department of Clinical and Community Pharmacy, College of Medicine and Health Sciences, An-Najah National University, Nablus, 44839 Palestine; WHO Collaborating Centre for Drug Information, National Poison Centre, Universiti Sains Malaysia (USM), Penang, 11800 Malaysia; Department of Pharmacology and Toxicology, College of Medicine and Health Sciences, An-Najah National University, Nablus, 44839 Palestine; PharmD Program, College of Medicine and Health Sciences, An-Najah National University, Nablus, Palestine

**Keywords:** Upper respiratory tract infections, Parents, Children, Antibiotic

## Abstract

**Background:**

In primary health care centres, upper respiratory tract infections (URTIs) in children are commonly encountered by physicians. Viruses cause most URTIs, but parents’ attitudes often represent an important reason for antibiotic abuse, which leads to the development and spread of antimicrobial resistance. The goal of this study was to examine parents’ knowledge, attitudes, and practices (KAP) about antibiotic use for children with URTIs in Palestine.

**Methods:**

A cross-sectional study was performed in primary health care centres in Nablus city from 1 June to 31 October 2012. A questionnaire was developed and administered to determine parents’ KAP regarding antibiotic use for their children with URTIs.

**Results:**

Three hundred and eighty-five parents completed the questionnaire. A total of 79.7 % of the parents were attentive to the truth that antibiotic misuse is responsible for bacterial resistance. Only 18.9 % of parents thought that antibiotics did not have any harmful side effects. Fifty nine per cent of parents did not agree that URTIs are mostly viral in origin and are self-limited. Almost 73 % of parents choose antibiotics as a treatment for URTIs, while earache (68 %) and fever (64 %) were the most common reasons for which parents expected antibiotics. However, more than 38 % of the parents never asked the paediatrician to prescribe antibiotics, and only 6 % congratulated their paediatricians for not prescribing antibiotics.

**Conclusions:**

Although there is a trusted relationship between parents and paediatricians, Palestinian parents have insufficient knowledge related to antibiotic use for URTIs in children, which results in inappropriate attitudes and practices. Educational interventions for both parents and physicians will reduce unnecessary antibiotic use and resistance.

**Electronic supplementary material:**

The online version of this article (doi:10.1186/s12887-015-0494-5) contains supplementary material, which is available to authorized users.

## Background

In primary health care centres, upper respiratory tract infections (URTIs) in children are commonly encountered by physicians [1–4]. These infections are considered the main cause of absenteeism from schools by children or from work by parents [5]. Furthermore, such infections in children bear a lot of economic burden on parents and healthcare system [5].

The World Health Organization, in its late report released in April 2014, revealed that antibiotic resistance is a serious and growing global problem [6]. Several studies reported the relationship between antibiotic use and the development of resistance [7–9]. Countries consuming the highest amounts of antibiotics have the highest rates of resistance [10]. Despite the fact that the majority of URTIs are viral in origin [11], antibiotic prescribing for URTIs is a common practice in paediatrics [12–14]. It is probable that 20–50 % of all antimicrobial use is medically inappropriate [15, 16]. Inappropriate prescribing of antibiotics is the most important reason behind the development of antibiotic resistance [17, 18].

The main contributors to the development of resistance in children are paediatricians and parents. Parental beliefs and expectations are important factors in determining whether an antibiotic is prescribed. When parents panic about acute illnesses, it leads to more frequent paediatric physician visits for URTIs and, subsequently, unnecessary antibiotic use [19–22]. Therefore, numerous reports have evaluated the factors related to antibiotic overuse. These factors consist of knowledge, attitudes and beliefs regarding antibiotic use [23–25], behaviours [26, 27], patient treatment satisfaction, patient-doctor communication, and patient experiences with antibiotics [25–28]. Proper public knowledge and attitude toward antibiotics is an important factor in rational antibiotic use and therefore minimizing development of antibiotic resistance [29]. Unfortunately, the pressure imposed on physicians to meet patients’ expectations is a major contributing factor for physicians to prescribe antibiotics for viral URTI [24, 30]. Therefore, parental knowledge, attitude and practice toward antibiotic use in URTI in their children is of great value [24, 31].

Many studies were conducted in Palestine regarding antibiotic misuse and purchasing antibiotics without a prescription [32, 33]. These studies evaluated the extent of storage and wastage of antibacterial agents in Palestinian households [34], self-medication with antibiotics [35], and patterns of parenteral antimicrobial prescription among paediatric hospitalised patients [36]. Neither of these studies, nor other studies conducted in Palestine, assessed parents’ knowledge, attitudes, and practices (KAP) regarding antibiotic use in URTIs in children. Thus, this study is the first of its kind in Palestine to evaluate parental KAP regarding antibiotic use in paediatrics. This study could provide baseline data for developing strategies for local health authorities’ educational purposes.

## Methods

### Study area and study design

A cross-sectional survey was performed in Primary Health Care (PHC) centres in selected areas in Nablus governorate. The study was carried out from June, 1st to the end of October in 2012. The PHC centre’s selection was based on geographic clustering sampling to obtain a representative sample of parents. Previously published studies about knowledge and attitudes toward antibiotics have been mostly carried out among physicians in primary healthcare centres [4, 37, 38]. For the purpose of this study, four primary health care centres were chosen. These centres have the followings in common which made them suitable as a study area [39, 40]: (1) they provide a full package of primary health care services; (2) they serve a large number of patients; and (3) they cover the three types of communities within this region (rural, urban, and Palestinian refugee camps). To the best of our knowledge, limited such studies were conducted on consumers such as parents [24, 31].

### Study population

The population of the study was the parents of children attending PHC centres aged between 18 and 50 years.

### Sampling procedure and sample size calculation

Sample size was calculated using a Raosoft sample size calculator. The calculation was based on 50 % response distribution, 5 % margin of error and 95 % confidence interval [41]. The assumption that the response rate is 50 % was based on the idea that both responses and response rates were completely unknown since there are no previously published similar studies from Palestine. The calculated sample size was 377. To ensure accuracy, the sample size was increased to 400 to account for any missing data or non-response rate. Ultimately, parents were selected using a convenience sampling method because it saves time, cost, and ease of accessibility to the researchers [42].

### Questionnaire development

A self-administered questionnaire was developed in Arabic after reviewing related studies [24, 31, 43]. Most of the developed questions were extracted from previously published validated studies in Greece and were tailored to suit the local situation and assure its applicability [31, 44]. The questionnaire is comprised of four main sections: demographic data related to participants; and knowledge, attitude, and practice concerning the use of antibiotics. Some items were added, and the questionnaire was modified to be used in the Palestinian setting. Content validity of the questionnaire was assured by a group of experts in the field of paediatrics, infectious diseases, clinical pharmacy and biostatisticians. A pilot study was conducted among 30 participants in order to check the clarity and readability of the questionnaire. The final version of the questionnaire was refined and corrected based on feedback from the participants.

The final questionnaire consisted of four sections (A, B, C, and D). Section A contained demographic data, including age, gender, education levels, residency, income, number of children, health insurance status and whether the child had a chronic disease such as asthma. Section B was adopted from Panagakou et al., [31] and included items concerning parental knowledge of antibiotics. Parents were requested to mark antibiotic names out of six frequently used medications in the Nablus district and to answer questions related to general antibiotic use, adverse effects and their use in viral infections. Furthermore, Section B explored sources of information regarding the use of antibiotics. Section “C” included items concerning parental attitudes toward antibiotics. Parents were asked for possible treatment options for paediatric URTI management. In addition, parents were asked specifically about the most serious symptoms that would have to be present in order for them to visit the paediatricians’ office. Other questions asked if parents thought that antibiotics were useful in relieving a variety of symptoms. Furthermore, parents were asked to indicate their expectations for antibiotic use corresponding to URTI symptoms and to designate the reasons for antibiotic use without medical advice. Finally, Section D illustrated parents’ answers to questions linked to the medical practice. Parents were asked to indicate if their paediatrician spends adequate time elucidating the illness and suggesting antibiotic treatment for a child’s illness, and if he/she is affected by their demand to prescribe antibiotics for their child. Parents were asked to answer the statements on a 5-point Likert scale (“strongly agree”, “agree”, “uncertain”, “disagree”, “strongly disagree” or “never”, “sometimes”, “often”, “most of the time”, “always”). A detailed description is provided in Additional file 1 about questions regarding knowledge, attitudes and practices of antibiotic use as an Arabic version.

### Ethical approval

The study was approved by the Palestinian Ministry of Health, and the institutional review board (IRB) of An-Najah National University (approval number 23-Apr 2012 on April 13, 2012) and verbal consent was obtained from survey participants. An written consent was waived according to the regulation of IRB.

### Statistical analysis

Data were entered and assessed with the Statistical Package for the Social Sciences (SPSS), version 16.0 for Windows. The analysis of answers for questions involved descriptive quantitative statistics, e.g., frequency and percentage for categorical variables and means ± standard deviation (SD) or medians (lower-upper quartiles) for numerical variables. The figures were created using Microsoft® Office Excel 2007.

## Results

Three hundred and eighty five questionnaires were collected back out of 400 that were initially distributed, giving a response rate of 96.2 %. The majority of respondents (62.6 %) were mothers with a mean age of 31.6 years (SD ± 7), and 74 % of parents considered their income as moderate. Approximately two-thirds of parents lived in the city. Nearly 43 % of participants had a university degree. The socio-demographic characteristics of respondents are shown in Table 1.

**Table 1:** Socio-demographic characteristics of the population studied (*n* = 385)

Variable	Frequency (%), or mean ± SD, or median [interquartile] *N* = 385
Gender	
Male	144 (37.4)
Female	241 (62.6)
Age ± SD (year)	31.6 ± 7
Median number of children aged less than six years	2 [1–2]
Median number of children aged more than six years	0 [0–2]
Health insurance	
Governmental insurance	142 (36.9)
Private insurance	127 (33.0)
Do not have insurance	116 (30.1)
Participant’s educational level	
Elementary school (primary)	79 (20.5)
High school (secondary school)	149 (36.4)
University	166 (43.1)
Income level of the family per month^a^	
Low (less than 500 JD)	67 (17.4)
Average (500–1000 JD)	285 (74)
High (more than 3000 JD)	33 (8.6)
Residency	
City	239 (62.1)
Rural	101 (26.2)
Palestinian refugee camps	45 (11.7)
Child with chronic disease	41 (10.6)

^a^1 Jordanian Dinar (JD) equals 1.41 US Dollar

### Knowledge

Most parents (61.6 %) stated that their physician was the main source of information regarding antibiotics, followed by their pharmacist (34.3 %); other sources, such as television, newspapers, and family members/friends accounted only for 2.8 % of parents’ sources of information. However, 1.3 % of parents stated they never received any information from any of these sources.

When parents were asked to discriminate between antibiotic products and other drugs, including analgesics, cough preparations and expectorants, and antipyretics, most parents (55.6 %) were able to identify that amoxicillin was an antibiotic, while only 8.1 and 3.1 % were able to identify that amoxicillin-clavulanic acid and cefuroxime, respectively, were antibiotics. Moreover, 24.2, 4.7 and 4.1 % of parents identified ibuprofen, paracetamol, and cough preparations, respectively, as antibiotics.

**Fig. 1:**
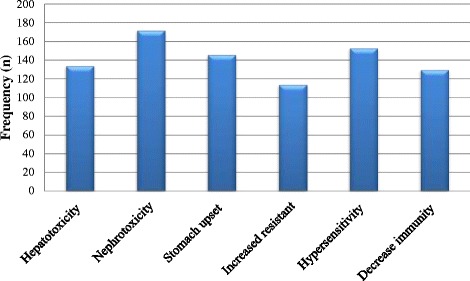
Parents’ knowledge regarding harmful adverse effects of antibiotics

Table 2 demonstrate the responses to questions related to knowledge. A total of 79.7 % of parents were attentive to the truth that antibiotic misuse is responsible for bacterial resistance, but 70.1 % would still give antibiotics to their child because they thought this would lead to a faster recovery. 59 % of parents did not agree that URTIs are mostly viral in origin and are self-limited without the need for antibiotic use. Only 18.9 % of parents thought that antibiotics did not cause any harmful side effects, while 78.1 % were certain that antibiotics might cause many harmful adverse effects (Fig. 1). Moreover, 71.7 % of parents thought that new stronger antibiotics are always emerging.

**Table 2:** Parental knowledge regarding antibiotic use in children with URTIs (*N* = 385). Questions adopted from Panagakou et al. [31]

Variable	Item	Frequency	Percentage %
Antibiotics can be used for any feverish child	Strongly agree	76	19.7
	Agree	153	39.7
	Disagree	110	28.6
	Strongly disagree	40	10.4
	Uncertain	6	1.6
Children with flu like symptoms get better faster when antibiotics are used	Strongly agree	69	17.9
	Agree	201	52.2
	Disagree	95	24.7
	Strongly disagree	15	3.9
	Uncertain	5	1.3
Most URTIs are viral in origin and are self-limited; thus, there is no need for antibiotic use	Strongly agree	43	11.2
	Agree	114	29.9
	Disagree	173	44.9
	Strongly disagree	37	9.6
	Uncertain	18	4.7
Antibiotics do not have any side effects	Strongly agree	19	4.9
	Agree	54	14.0
	Disagree	163	42.3
	Strongly disagree	138	35.8
	Uncertain	11	2.9
Inappropriate use of antibiotics reduces their efficacy and drives bacterial resistance	Strongly agree	126	32.7
	Agree	181	47
	Disagree	53	13.8
	Strongly disagree	15	3.9
	Uncertain	10	2.6
Antibiotic use can prevent complications from URTIs	Strongly agree	68	17.7
	Agree	223	57.9
	Disagree	63	16.4
	Strongly disagree	12	3.1
	Uncertain	19	4.6
Scientists can produce new antibiotics for resistant bacteria	Strongly agree	96	24.4
	Agree	180	46.8
	Disagree	27	7.0
	Strongly disagree	18	4.7
	Uncertain	64	16.6

### Attitude and expectation

**Fig. 2:**
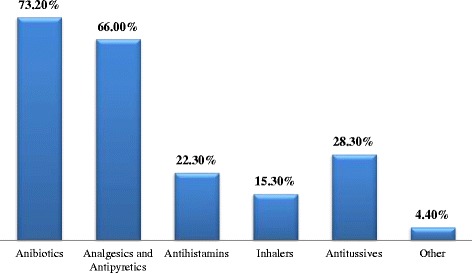
Parental expected treatment for paediatric URTIs

When parents were given possible treatment options for the management of paediatric URTIs, more than two-thirds (73.2 %) of participants chose antibiotic therapy, and 66 % of parents choose analgesics and antipyretics as a possible treatment options for URTIs, while only 15.3 % of participants choose inhalers as a possible therapy (Fig. 2). In addition, when parents were asked specifically about the most serious symptoms that would have to be present in order for them to visit the paediatricians office (including fever, runny nose, cough, sore throat, ear pain, and change in behaviour) in the case of URTIs, 78.4 and 44.7 % of symptoms were fever and ear pain, respectively, often accompanied by other symptoms.

**Fig. 3:**
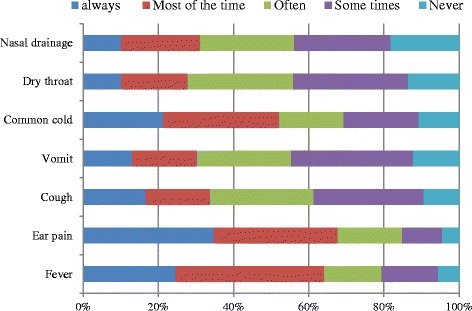
Parental expectations for antibiotic use corresponding to upper respiratory tract infection symptoms. Questions adopted from Panagakou et al. [31]

Few parents would ask their paediatrician for antibiotics for nasal drainage (22 %) or dry throat (11 %), while the majority of parents would want their paediatrician to recommend an antibiotic if their child had an earache (68 %), fever (64 %), cold (52 %), cough (34 %), or was vomiting (30 %). Figure 3 shows parental expectations for antibiotic use corresponding to URTI symptoms. There are many reasons for parents to administer antibiotics to their children without having received previous medical advice. In particular, 24.7 % of parents used antibiotics as self-medication due to economic hardships or lack of time, while 50.6 % would give antibiotics to their child because they believed that symptoms (e.g., earache, fever, cold, cough) were not dangerous as much as necessary to see the paediatrician.

**Fig. 4:**
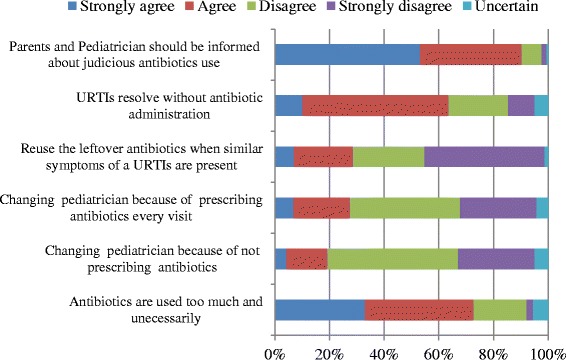
Percentage of parents’ responses to questions related to attitude. Questions adopted from Panagakou et al. [31]

Figure 4 indicates parental attitudes for antibiotic use in URTIs. The majority of parents (72.7 %) agreed that antibiotics are extensively used without proper indications and affirmed that they would not change paediatricians if they did not easily prescribe antibiotics (76 %); however, 27 % declared that they would change paediatricians because they easily prescribed antibiotics. About 63.5 % of parents agreed that it was better to keep away from the use of antibiotics to their child for simple or uncomplicated URTIs.

### Practice

**Fig. 5:**
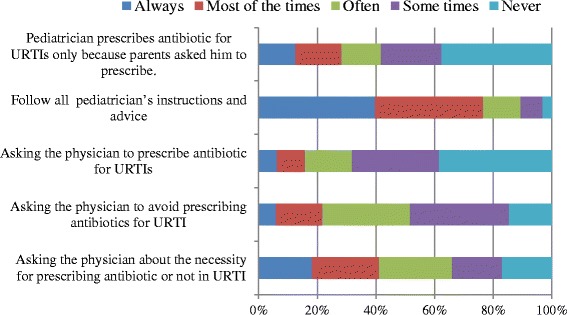
Percentage of parents’ responses to questions related to practice. Questions adopted from Panagakou et al. [31]

Figure 5 illustrates parents’ responses to questions related to practice. More than 38 % of parents declared that they never asked their paediatrician to prescribe antibiotics, while only 6 % of parents congratulated paediatricians for not prescribing antibiotics. However, about 41.1 % of parents would ask the paediatrician whether antibiotic administration was necessary. About 28.3 % of parents thought that their paediatrician prescribed antibiotics based on their request, and, more notably, 76.6 % declared they exactly follow paediatricians’ directions.

## Discussion

The current study aimed to analyse knowledge and attitudes concerning antibiotic use and practices in the management of childhood URTIs in a large sample of parents. The issue of the current study is novel in the Palestinian population. It is considered a part of social science that often lags behind.

Our study demonstrated that Palestinian parents and paediatricians have a trusting relationship; the great majority of parents have confidence in the information and prescriptions supplied to them by paediatricians, and only a few parents would change paediatricians if they over- or under-prescribed antibiotics in the case of URTIs in children. Furthermore, 76.6 % stated that they precisely follow paediatricians’ recommendations, and almost two-thirds of parents indicated their paediatricians as the main source of information about use or misuse of antibiotics. Xiang et al. [45] identified media (e.g., television) as the main source of such information about use or misuse of antibiotics, despite the fact that similar KAP studies reported paediatricians as the preferred source of information [43].

Most of the Palestinian parents were attentive to the truth that antibiotic misuse is responsible for bacterial resistance, although 59 % of them did not agree that URTIs are mostly viral in origin and are self-limited without the need for antibiotic use, which is in contrast to Greek parents, of which 80 % believed that URTIs are mostly self-limited [31]. It is inaccurate to suppose that 59 % of the parents preferred only antibiotic treatment because most of them also favoured other medications indicated for symptom relief such as analgesics, antipyretic, antihistamines, inhalers, and cough preparations.

Our results demonstrated that fever symptoms accompanied with URTIs were the most common reason for a paediatric visit in which parents would expect to receive antibiotics. Similarly, a cross-sectional KAP study involving 421 parents in Malaysia showed that 76 % of parents believed that antibiotics were helpful in the treatment of fever [24].

The use of “leftover” and “shared” antibiotics by parents to their child are common situations in the Palestinian and Malaysian communities; 27.6 % of Palestinian parents reused the leftover and shared antibiotics between their children, while 15 % of Malaysian parents reused leftovers and 24 % shared antibiotics. Parents believed that their child complained of the same illnesses because they had similar symptoms, therefore they would give the leftover antibiotics and shared it with others, and only bring their children to paediatrician if there was no improvement [24]. Interestingly, almost 51 % of parents choose antibiotics as the first choice of treatment for URTIs despite 78 % of them understanding that antibiotic use is associated with harmful and adverse effects on body systems, especially nephrotoxicity and hepatotoxicity.

Furthermore, 72.7 % of parents in the current study believed that antibiotics were used too much and unnecessarily. Comparable findings were found in previous similar studies such as Panagakou et al. [31] (78 %) and Rouusounides et al. [43] (81 %). This could be somewhat contributed to by paediatricians’ behaviours and their antibiotic prescribing practices in Palestine [35, 36, 46, 47], which is one of the developing countries in the Middle East that has a high antimicrobial resistance rate [32, 48, 49]. There were several factors that may have induced inappropriate antibiotic prescription, including diagnostic uncertainty, socio-cultural and economic pressures, lack of knowledge, and fear of litigation [50, 51]. A more recent study demonstrated that many general practitioners had a moderate level of knowledge concerning the management of URTIs [4].

### Strengths and limitations of the study

This is the first study that was conducted to assess parental KAP on antibiotic use in URTIs in Palestine. In addition, the study response rate of 96.1 % is considered reasonable for a community survey. Nevertheless, there were some limitations of this study. These limitations were associated with using a convenience sample, which might not be representative of the whole community in Palestine. Furthermore, the data were collected from parents attending PHC centres which limit the generalizability of the results to other types of health care services such as private sectors. While efforts were made to obtain representative samples, the over representation of PHC and higher educational level in the study sample might indicate a possible selection bias. Another limitation is that parents were asked several questions about their experience and antibiotic use in the past, which may lead to recall bias. Lastly, small sample size of the subgroups made comparative analyses to be problematic. For example, comparison between those living in refugee camps and city residents would have been helpful in identifying the most appropriate groups to target with educational programmes.

## Conclusions and recommendations

In conclusion, we found that Palestinian parents’ lack of knowledge on antibiotic use for paediatric URTIs resulted in inappropriate attitudes and practices. On the other hand, there is a trusted relationship between parents and paediatricians, and there is confidence in the information and prescriptions provided to them from doctors: only a few parents would change their paediatrician according to antibiotic prescription patterns. However, parents also believed that inappropriate use of antibiotics reduces their efficacy and drives resistance. Unfortunately, a large number of parents did not agree that URTIs are mostly viral of origin; parents’ also self-limited antibiotic use, and three-fourths expected antibiotics to be a choice for paediatric URTI treatment. Educational interventions for both parents and physicians will reduce unnecessary antibiotic use and resistance. Strengthening and application of pharmacy regulations related to the over-the-counter sale of antibiotics is needed in community pharmacies.

## Additional file

Additional file 1:
**Study questionnaires.** This is the final version of the Arabic version that was used for assessing parents’ knowledge, attitudes, and practices regarding antibiotic use in upper respiratory tract infections in children. (DOCX 36 kb)

## Abbreviations

URTIs: Upper respiratory tract infections
KAP: Knowledge, attitudes, and practices
PHC: Primary health care
SD: Standard deviation
IRB: Institutional review board
