# Supplementary material for: Parental knowledge, attitudes and practices regarding antibiotic use for acute upper respiratory tract infections in children: a cross-sectional study in Palestine
Source: BMC Pediatr. 2015 Nov 11;15:176. doi: 10.1186/s12887-015-0494-5 (PMC4642624; doi:10.1186/s12887-015-0494-5)
Supplement: Additional file 1: — Study questionnaires. This is the final version of the Arabic version that was used for assessing parents’ knowledge, attitudes, and practices regarding antibiotic use in upper respiratory tract infections in children. (DOCX 36 kb) [file 12887_2015_494_MOESM1_ESM.docx]

**Additional file 1.** **Study questionnaires. This is the final version of the Arabic version that was used for assessing parents' knowledge, attitudes, and practices regarding antibiotic use in upper respiratory tract infections in children.**

**معرفة وموقف الآباء والأمهات من استخدام المضادات الحيوية لمعالجة التهابات الجهاز التنفسي العلوي عند أطفالهم**

هذا الاستبيان يقوم به مجموعة من الباحثين من جامعة النجاح الوطنية لغرض دراسة علمية ويهدف لقياس مدى معرفة الآباء والأمهات عن استخدام المضادات الحيوية لمعالجة التهابات الجهاز التنفسي العلوي عند أطفالهم وموقفهم منها.المعلومات ستعامل بسرية تامة و لن يتم التعرض لخصوصيات المريض أو اسمه و ستستخدم لأغراض البحث العلمي فقط. المشاركة في هذه الدراسة اختيارية و تطوعية, نرجو من حضرتكم التكرم بالإجابة عن جميع الأسئلة التالية بدقة شاكرين لكم حسن تعاونكم.

- **العمر** (للمشترك/ة): **.........................**
- **الجنس:**

| 1. ذكر | 1. انثى |  |
| --- | --- | --- |

- **مكان السكن الأصلي:**

| 1. مدينة | 1. قرية | 1. مخيم |
| --- | --- | --- |

- **المستوى التعليمي للأب:**

| 1. ابتدائي أو إعدادي | 1. ثانوية عامة | 1. مستوى جامعي |
| --- | --- | --- |

- **المستوى التعليمي للأم:**

| 1. ابتدائي أو إعدادي | 1. ثانوية عامة | 1. مستوى جامعي |
| --- | --- | --- |

- **مستوى الدخل للاسرة:**

| 1. منخفض (اقل من 500 دينار) | 1. متوسط (500-1000 دينار) | 1. مرتفع (أكثر من 1000 دينار) |
| --- | --- | --- |

- **تأمينك الصحي:**

| 1. تأمين حكومي | 1. تأمين خاص | 1. لا يوجد لدي تأمين صحي |
| --- | --- | --- |

- **برأيك يعد حصولك على الخدمات الصحية:**

| 1. سيء | 1. متوسط | 1. جيد |
| --- | --- | --- |

- **هل يعمل الأبوين** **في المجال الطبي ؟**

| \| 1. نعم \| 1. لا \|  \| \| --- \| --- \| --- \| |  |
| --- | --- | --- | --- | --- |

- **عدد الاطفال الأقل من 6 سنوات:.........**

عمر الطفل الأول ___ عمر الطفل الثاني ___ عمر الطفل الثالث ___

**عدد الأطفال الأكثر من 6 سنوات:.................**

- **هل يعاني احد أطفالك من الأمراض المزمنة المتعلقة بالجهاز التنفسي (الربو مثلا ) ؟**
- نعم
- لا

| 1. نعم | 1. لا |  |
| --- | --- | --- |

**================================================================**

- **المصدر الأول للمعلومات التي ترشدك عن كيفية استخدام المضادات الحيوية بحكمة:**

| 1. الطبيب | 2) الصيدلاني | 1. الراديو والتلفاز |
| --- | --- | --- |
| 1. الصحف والمجلات | 1. الأقارب والأصدقاء | 1. أخرى ..................... |

- **برأيك أي من الأدوية التالية هو مضاد حيوي:**

| 1. تروفين 2. اموكسيكير , موكسيبين , اموكسيتيد ,,,, 3. اكامول 4. بولمادرين , برافلو , توسيبال ,,,, 5. اوجمينتين , اوجمين ,,, 6. زينات , زينيكسيم ,,, |
| --- |

| 1) نعم | 1. لا |  |
| --- | --- | --- |

- **تعد المضادات الحيوية الخيار الأول والأنجع لعلاج التهابات الجهاز التنفسي العلوي عند الأطفال:**
- **مقياس معرفة الوالدين فيما يتعلق بالمضادات الحيوية واستخدامها لمعالجة التهابات الجهاز التنفسي عند الأطفال**

|  | **أوافق بشدة** | **أوافق** | **أعارض** | **أعارض بشدة** | **لا رأي لي** |
| --- | --- | --- | --- | --- | --- |
| 1. **يجب إعطاء المضادات الحيوية لجميع الأطفال عندما يصابون بالحمى (ارتفاع درجة الحرارة)** |  |  |  |  |  |
| **2- الأطفال الذين يعانون من أعراض الأنفلونزا يتحسنون بشكل أسرع عندما يتم إعطاؤهم المضادات الحيوية** |  |  |  |  |  |
| **3- التهابات الجهاز التنفسي غالبا ما يكون سببها فيروسي ولا تحتاج الى مضادات حيوية** |  |  |  |  |  |
| **4- المضادات الحيوية ليس لها أي مضاعفات جانبية** |  |  |  |  |  |
| **5- الاستخدام المفرط للمضادات الحيوية يقلل من فعاليتها ويقود إلى مقاومة الجراثيم.** |  |  |  |  |  |
| **6- استخدام المضادات الحيوية يمكن من منع المضاعفات الناجمة عن التهابات الجهاز التنفسي العلوي.** |  |  |  |  |  |
| **7- سيتمكن العلماء من إنتاج مضادات حيوية قادرة على معالجة أنواع البكتيريا المقاومة للمضادات الحيوية المتوافرة حاليا** |  |  |  |  |  |

**==================================================================**

- **إذا كان طفلك يعاني من أعراض التهابات الجهاز التنفسي العلوي (سيلان الأنف، التهاب الحلق والقيء والسعال والحمى) ما عدد الأيام التي تمضيها قبل اصطحابه إلى طبيب الأطفال؟ ............. يوما.**

| 1) حمى | 2) سيلان الأنف |  |
| --- | --- | --- |
| 3) احتقان الحلق | 4) بحة في الصوت |  |
| 5) الم الأذن | 6) تغيير السلوك | 7) أعراض أخرى .................. |

- **أي من الأعراض التالية تجعلك تلجأ لطيب الأطفال؟ (ممكن اختيار أكثر من اجابة)**

| 1) المضادات الحيوية | 2) مسكنات الألم وخافضات الحرارة |  |
| --- | --- | --- |
| 3) أدوية الحساسية | 4) بخاخات الأنف | . |
| 5) أدوية السعال | 6) أعراض أخرى ................ |  |

- **ما هو نوع العلاج الذي تتوقع من طبيب الأطفال اقتراحه لطفلك عندما يعاني من التهابات الجهاز التنفسي العلوي؟ (ممكن اختيار أكثر من اجابة)**

- **ما مدى رغبتك في وصف المضاد الحيوي لطفلك عندما يعاني من الأعراض التالية:**

|  | **دائما**  **95-100%** | **معظم الوقت**  **70- 95%** | **غالبا**  **30-70%** | **أحيانا**  **5-30%** | **مطلقا**  **0-5%** |
| --- | --- | --- | --- | --- | --- |
| **نزلات البرد** |  |  |  |  |  |
| **سيلان الأنف** |  |  |  |  |  |
| **جفاف الحلق** |  |  |  |  |  |
| **السعال** |  |  |  |  |  |
| **التقيؤ** |  |  |  |  |  |
| **الحمى** |  |  |  |  |  |
| **الم في الأذن** |  |  |  |  |  |

| 1) ايذاء الكبد | 2) ايذاء الكلية |  |
| --- | --- | --- |
| 3) إيذاء المعدة | 4) زيادة مقاومة الجراثيم للمضادات الحيوية |  |
| 5) الحساسية | 6) ضعف جهاز المناعة |  |
| 7) ليس لها اي مخاطر | 8)مخاطر أخرى............................. |  |

- **برأيك ما هي مخاطر المضادات الحيوية؟ ( ممكن اختيار أكثر من إجابة)**

**==============================================================**

- **اي من الأمور التالية تؤدي الى إعطاء طفلك المضادات الحيوية دون استشارة طبيب الأطفال ؟( ممكن اختيار أكثر من إجابة)**

| 1) لأنه لم يكن لديك الوقت الكافي لزيارة الطبيب, أو عدم توفر المال الكافي |
| --- |
| 2) لأنك كنت تعتقد أن حالة طفلك غير خطيرة |
| 3) لان طبيب الأطفال وصف نفس الدواء سابقاً لطفلك و لنفس الأعراض الحالية |
| 4) لأن الصيدلاني أوصى بمضاد حيوي معين |
| 5) لان احد الأقارب أو الجيران أوصى بإعطاء مضاد حيوي معين |

- **الرجاء الإجابة على الأسئلة التالية بوضع إشارة x في المكان المناسب**

|  | **دائما**  **95-100%** | **معظم الوقت**  **70- 95%** | **غالبا**  **30-70%** | **أحيانا**  **5-30%** | **مطلقا**  **0-5%** |
| --- | --- | --- | --- | --- | --- |
| **1- هل تسأل الطبيب عن ضرورة أو عدم ضرورة وصف المضاد الحيوي لطفلك ؟** |  |  |  |  |  |
| **2- هل ترغب بعدم وصف الطبيب للمضادات الحيوية لطفلك ؟** |  |  |  |  |  |
| **3- هل تطلب من الطبيب بشكل مباشر أن يصف لطفلك المضاد الحيوي ؟** |  |  |  |  |  |
| **4- هل تتبع تماما جميع تعليمات ونصائح طبيب الأطفال عند استخدام المضاد الحيوي ؟** |  |  |  |  |  |
| **5- هل تعتقد أن طبيب الأطفال يصف لطفلك المضاد الحيوي فقط لأنك أنت طلبت وصفه؟** |  |  |  |  |  |

- **مقياس رغبة وسلوك الوالدين فيما يتعلق بالمضادات الحيوية واستخدامها لمعالجة التهابات الجهاز التنفسي عند الأطفال**

| **الرجاء الإجابة على الأسئلة التالية بوضع إشارة x في المكان المناسب** | **أوافق بشدة** | **أوافق** | **أعارض** | **أعارض بشدة** | **لا رأي لي** |
| --- | --- | --- | --- | --- | --- |
| **1- هل تعتقد بأنه يتم استخدام المضادات الحيوية أكثر من اللازم وبشكل غير ضروري؟** |  |  |  |  |  |
| **2- هل قمت بتغيير طبيب أطفالك لأنه لم يقم بوصف المضادات الحيوية لطفلك كما تريد؟** |  |  |  |  |  |
| **3- هل قمت بتغيير طبيب أطفالك لأنه في كل زيارة يقوم بوصف المضادات الحيوية ؟** |  |  |  |  |  |
| **4- هل تقوم بإعطاء طفلك بقايا المضاد الحيوي السابق عندما يصاب بنفس الأعراض السابقة ؟** |  |  |  |  |  |
| **5- هل تعتقد أن معظم التهابات الجهاز التنفسي العلوي يمكن أن تحل من دون استخدام المضادات الحيوية لأنها تزول تلقائياً ؟** |  |  |  |  |  |
| **6- هل تعتقد أنه ينبغي إبلاغ الآباء وأطباء الأطفال عن استخدام المضادات الحيوية بحكمة ؟** |  |  |  |  |  |

**مع الشكر الجزيل لوقتكم وتعاونكم**
